# PD-L1 Under Regulation of miR-429 Influences the Sensitivity of Gastric Cancer Cells to TRAIL by Binding of EGFR

**DOI:** 10.3389/fonc.2020.01067

**Published:** 2020-07-22

**Authors:** Jinqi Lv, Tianshu Guo, Xiujuan Qu, Xiaofang Che, Ce Li, Shuo Wang, Jing Gong, Peihong Wu, Yang Liu, Yunpeng Liu, Ling Xu

**Affiliations:** ^1^Department of Medical Oncology, The First Hospital of China Medical University, Shenyang, China; ^2^Key Laboratory of Anticancer Drugs and Biotherapy of Liaoning Province, The First Hospital of China Medical University, Shenyang, China; ^3^Liaoning Province Clinical Research Center for Cancer, Shenyang, China; ^4^Key Laboratory of Precision Diagnosis and Treatment of Gastrointestinal Tumors, Ministry of Education, Shenyang, China

**Keywords:** gastric cancer, TRAIL, PD-L1, miR-429, EGFR

## Abstract

Tumor necrosis factor-related apoptosis-inducing ligand (TRAIL) has received extensive attention as a cancer therapeutic due to its high propensity for tumor targeting with minimal toxicity to healthy tissue. Gastric cancer (GCa) cells show high levels of TRAIL resistance. Epidermal growth factor receptor (EGFR) antagonizes TRAIL-induced apoptosis, but the mechanisms of these effects remain unclear. Our past research confirmed TRAIL-resistant (BGC823 and SGC7901) and TRAIL-sensitive cells (HGC27 and MKN45). miR-429 associated with TRAIL sensitivity was screened using microRNA arrays. The transfection of mimics and inhibitors confirmed that miR-429 negatively correlated with GCa TRAIL resistance. The target gene of miR-429 was identified as PD-L1, which positively correlated with TRAIL resistance through gene silencing and recovery experiments. Using co-immunoprecipitation (co-IP) and proximity ligation assay, we demonstrated that the pro-survival effects of PD-L1 are mediated through the binding and activation of EGFR. Cell viability experiments demonstrated that PD-L1 is key to the maintenance of cell viability in TRAIL-treated cells. This indicated that PD-L1 binds to and participates in EGFR activation through miR-429 regulation to antagonize TRAIL-induced apoptosis. This provides a new theoretical basis for the combination of the EGFR monoclonal antibodies including cetuximab, PD-L1 inhibitors, and human recombinant TRAIL in gastric cancer therapy and can filter patients who are currently sensitive to TRAIL treatment.

## Introduction

Gastric cancer (GCa) remains prevalent across the globe for which current treatment strategies are lacking. In metastatic disease, the outcomes are poor, with median survival rates of ~1 year (1). Tumor necrosis factor-related apoptosis-inducing ligand (TRAIL) is a member of the TNF superfamily (TNFSF) that acts as an agonist for DR4 and DR5 to transfer extracellular death signals for cancer therapy (2). TRAIL has received extensive attention as a cancer therapeutic due to its high propensity for tumor targeting with minimal toxicity to healthy tissue (3, 4). However, in the clinic, the effectiveness of TRAIL has been suboptimal primarily due to (5) tumor resistance, the mechanisms of which are incompletely understood. The ability to overcome TRAIL resistance would represent a step change in the treatment of GCa (6, 7).

MicroRNAs (MiRNAs) degrade messenger RNA (mRNA) through the targeting of the 3′-UTRs of cellular mRNAs (8). Of note is that several miRNAs regulate TRAIL resistance in tumor cells (9–12). The miRNAs associated with TRAIL resistance in GCa are, however, undetermined (13). Recent studies have shown that miR-429 is poorly expressed in gastric cancer and is related to the epithelial–mesenchymal transition (EMT) processes of gastric cancer (14). We speculate that miR-429 may therefore be related to TRAIL resistance.

Recent studies suggest that TRAIL induces epidermal growth factor receptor (EGFR) activation and contributes to drug resistance in TRAIL-resistant GCa (15). A variety of molecules can promote or inhibit TRAIL-induced EGFR activation and, thus, affect sensitivity to TRAIL. Examples include SRC/CAV-1, cbl-b, and FLIP (15, 16), which participate in TRAIL-induced EGFR activation. Here, we assessed PD-L1 expression in GCa cells resistant to TRAIL therapy. PD-L1 mediates the malignant phenotypes of tumors through its ability to promote chemoresistance (17). Studies have shown that, in lung cancer, PD-L1 expression positively correlates with the degree of EGFR phosphorylation (18). We herein report that the miR-429/PD-L1 axis regulates EGFR activation to promote TRAIL resistance. The current therapeutic combination of optimized TRAIL agonists and the recently discovered powerful apoptotic sensitizers represents a promising strategy for clinical treatment. The most potent TRAIL sensitizers are CDK9 inhibitors, which greatly enhance the sensitivity of tumor cells to TRAIL (19). This study also reveals a new TRAIL resistance mechanism that provides a new theoretical basis for the co-therapy of the EGFR monoclonal antibody cetuximab, PD-L1 inhibitors, and human recombinant TRAIL in GCa cases.

## Methods

### Cell Lines and Treatments

The BGC823, SGC7901, HGC27, and MKN45 cell lines were purchased from Shanghai Chinese Academy of Sciences (Shanghai, China). Cells were grown in RPMI 1640 plus 10% fetal bovine serum (FBS) and 1% cyan/streptomycin (all obtained from Gibco) under standard cell culture conditions. The cell lines were tested monthly for mycoplasma and passed STR-DNA tests. Cells were passaged ≤12 generations. Recombinant human TRAIL was purchased from Peprotech, 100 ng/ml for cell culture and experiment.

### MicroRNA Array Analysis

MicroRNA arrays were purchased from Affymetrix (Thermo Fisher Scientific) and used to screen the differentially expressed miRNAs in both TRAIL-resistant and TRAIL-sensitive cells (MKN45 vs. BGC823).

### Transfections

Small interfering RNAs (siRNAs) targeting PD-L1, PD-L1 complementary DNA (cDNA) plasmids, and miR-429 mimics or inhibitors (RIBOBIO) were transfected into the indicated GCa lines using Lipofectamine 3000 (see Supplementary Table 1 for sequences). Mimics were synthesized using chemical synthesis to enhance the function of endogenous miRNAs. miRNA inhibitors are chemically modified inhibitors that target specific miRNAs in cells. The transfection efficiencies were verified by quantitative PCR (qPCR) and Western blot analysis.

### Bioinformatics

Survival analysis and gene expression were assessed from GCa datasets using Kaplan–Meier (KM) plots (20) (kmplot.com, OS, auto-select best cutoff) and GEPIA2 (21) (http://gepia2.cancer-pku.cn/) online analytics. GCa and paracancerous databases included The Cancer Genome Atlas (TCGA) and Genotype-Tissue Expression (GTEx). Gene expression cutoff values were assessed in survival analysis. All possible cutoff values between the lower and upper quartiles were computed, and the best-performing thresholds were used as the cutoff values. MiR binding was predicted *via* Targetscan and the UCSC genome.

### FACs Analysis

Cells (1 × 10^5^) were detached and co-stained with annexin V/propidium iodide (PI) (BD Bioscience). Apoptotic cells that were positively stained were assessed on a BD LSR Fortessa flow cytometer and analyzed using FlowJo V10 software.

### Western Blot Analysis

Western blots were performed as previously described (22). Protein bands were visualized using ImageJ. The primary antibodies used were as follows: PD-L1 (1:1,000; 66248-1-Ig, Proteintech, China), GAPDH (1:1,000; 10494-1-AP, Proteintech, China), cleaved caspase-3 (1:1,000; CST,9664, USA), EGFR (1:1,000; CST,4267, USA), P-EGFR-Tyr1068 (1:1,000; CST,3777, USA), AKT (1:1,000; CST,4691, USA), P-AKT-Ser473 (1:1,000; CST,4060, USA), mTOR (1:1,000; CST,2983, USA), P-mTOR-Ser2448 (1:1,000; CST,5536, USA), DR4 (1:1,000; CST,42533, USA), and DR5 (1:1,000; CST,8074, USA). These antibodies are used to detect the expressions of these proteins.

### Quantitative PCR

Total RNA was extracted (TaKaRa) with the stem-loop method and used for cDNA synthesis and miRNA quantitative assessments. First-strand cDNA was obtained *via* reverse transcription (Thermo Fisher) and quantified by qPCR on a QuantStudio 3. The values were normalized to β-actin and calculated using the 2^−DDCt^ method. All primers are shown in Supplementary Table 1.

### Immunofluorescence Analysis

Immunofluorescence was performed as previously described (22). The cells were probed with anti-PD-L1 to assess its total levels and cellular localization.

### Co-IP Assays

Co-immunoprecipitation (co-IP) assays were performed as per our previous studies (22). PD-L1 was precipitated with antibodies or control IgG following pre-clearing of the lysates with protein G-agarose beads for 6 h. The immunoprecipitates were washed (four times) in lysis buffer and Western blot analysis was performed using anti-phosphorylated EGFR (p-EGFR) and EGFR antibodies.

### Cell Viability Assessments

The transfected cells were plated in 96-well plates (1 × 10^3^ cells per well) and TRAIL treated for 6 days. The blank medium was used as the control. Three pairs of parallel holes were conducted in each experiment. Cell viability was assessed each day using cell viability kits (Promega, G7570) according to the manufacturer's instructions.

### TUNEL Assay

Terminal deoxynucleotidyl transferase UTP nick-end labeling (TUNEL) assays were performed to detect apoptosis. Transfected cells were plated onto coverslips, treated with TRAIL, paraformaldehyde fixed, and permeabilized with Triton X. The cells were blocked in 3% H_2_O_2_ in the dark and TUNEL-stained for 90 min. The cell nuclei were counterstained with DAPI. Cells were imaged on a BX51 microscope.

### Luciferase Assays

Wild type (WT; mutant) 3′-UTR of PD-L1 mRNAs with miR-429 binding sites were cloned to luciferase plasmids (Supplementary Figure 1E). The plasmids were synthesized by OBiO. Mimic-429, Renilla plasmids, and the luciferase constructs were transfected into cells for 48 h and the luciferase activity was assessed.

### *In situ* Proximity Ligation Assay

Duolink *in situ* proximity ligation assay (PLA; Olink Bioscience) was used to detect the interactions of PD-L1 and p-EGFR (23). Immunofluorescence was performed as previously described (22). Oligonucleotide-conjugated PLA probe antibodies were directed against the primary antibodies for PD-L1 and p-EGFR. Annealing of the PLA probe occurred when PD-L1 and p-EGFR were in close proximity, which initiates the amplification of repeat sequences recognized by the fluorescently labeled oligonucleotide probe. For detection, Duolink detection kit 563 was used. The samples were imaged *via* confocal fluorescence microscopy (FV1000S-SIM/IX81, Japan).

### Statistics

Data are the mean ± *SD* (*n* = 3). Group comparisons were performed using Student's two-tailed *t*-test *via* the SPSS Statistics 17.0.1 package. Spearman's correlation analysis was used to analyze the correlation between mir-429 and PD-L1 mRNAs. *P* < 0.05 indicated significant difference between groups.

## Results

### miR-429 Expression Correlates With the TRAIL Sensitivity of GCa Cells

**Figure 1:**
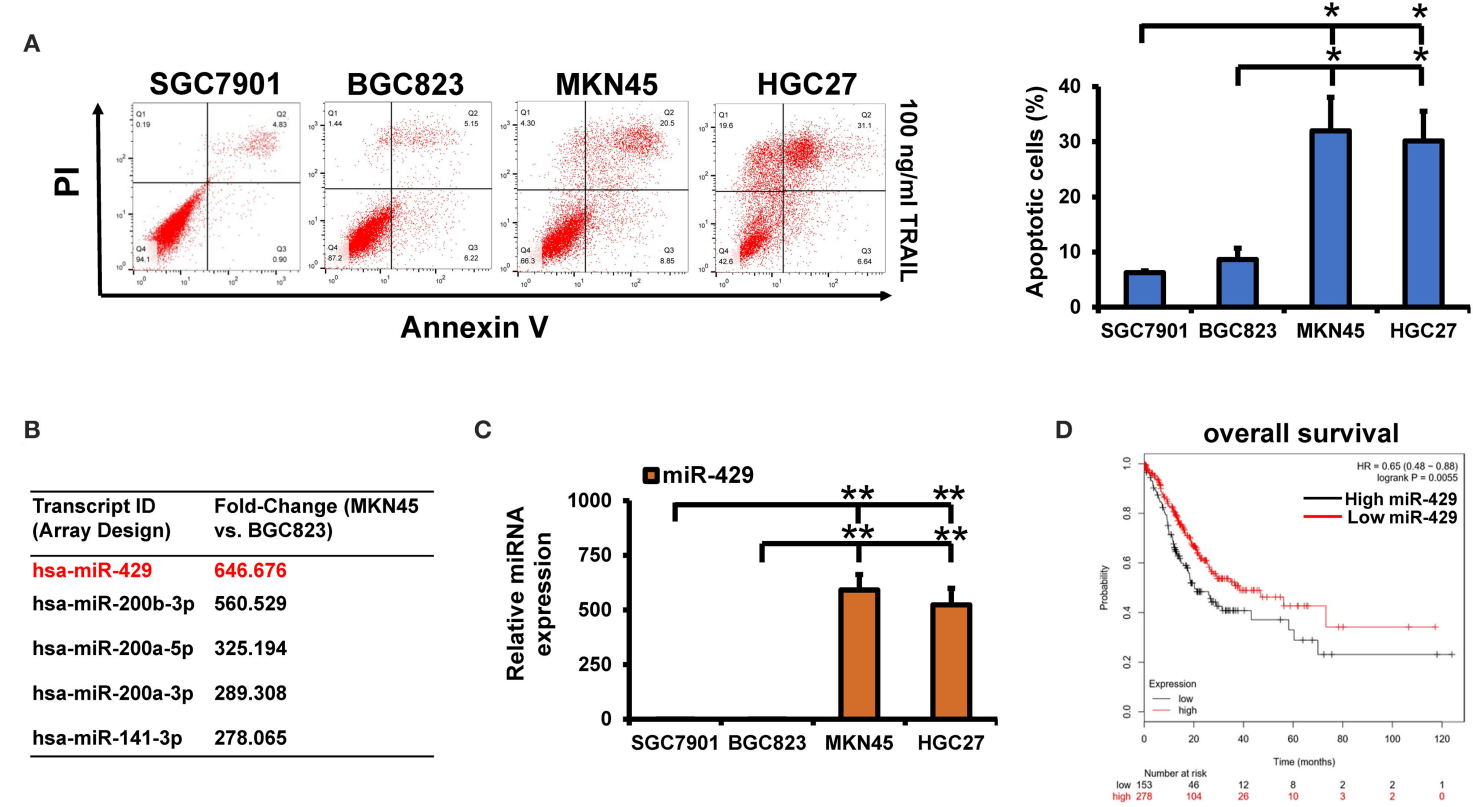
Screening for differential microRNAs (miRNAs) in tumor necrosis factor-related apoptosis-inducing ligand (TRAIL)-sensitive and TRAIL-resistant gastric cancer (GCa) cells. **(A)** Apoptosis was assessed by flow cytometry following TRAIL treatment (100 ng/ml). **(B)** Differentially expressed miRNAs in the MKN45 and BGC823 cell lines using microRNA array. **(C)** miR-429 expression in GCa cells assessed through quantitative PCR (qPCR). **(D)** Online analysis of miR-429 on the prognosis of GCa through Kaplan–Meier (KM) plots. Results are the mean ± SEM of triplicate samples from three independent experiments. *P < 0.05, **P < 0.01.

GCa cells show variable sensitivity to TRAIL (22). We stained cells with annexin V and PI and apoptosis was assessed in the GCa cell lines following TRAIL treatment. We found that BGC823 and SGC7901 cells were TRAIL-resistant while HGC27 and MKN45 were TRAIL-sensitive (Figure 1A). To rule out the effects of TRAIL receptor expression on TRAIL sensitivity, DR4 and DR5 expressions were assessed (Supplementary Figure 1A), for which no differences were observed across cell types. Differentially expressed miRNAs in resistant *vs*. sensitive cells have been assessed using microRNA array (Figure 1B). The raw data of the microRNA array have been uploaded to the ArrayExpress database (ID: E-MTAB-9151). In BGC823 and MKN45 cells, significant differences in miR-429, miR-200a-5p, miR-200b-3p, miR-200a-3p, and miR-141-3p were observed. miR-429 was selected for further analysis. We further verified the effects of TRAIL treatment on miR-429 expressions in all cell lines by qPCR (Figure 1C). miR-429 was highly expressed in TRAIL-sensitive lines, but poorly expressed in resistant cells, suggesting its correlation with the sensitivity of GCa cells to TRAIL. miR-429 expression further declined following TRAIL treatment in resistant cells, but had little effect in TRAIL-sensitive HGC27 and MKN45 cells (Supplementary Figure 1C). Upon assessment of the effects of miR-429 on the prognosis of patients with GCa, those with low miR-429 expressions displayed poor prognosis (Figure 1D), suggesting that miR-429 regulates the malignant progression of GCa and mediates TRAIL sensitivity.

### miR-429 Inhibition in HGC27 and MKN45 Decreases Their Sensitivity to TRAIL

**Figure 2:**
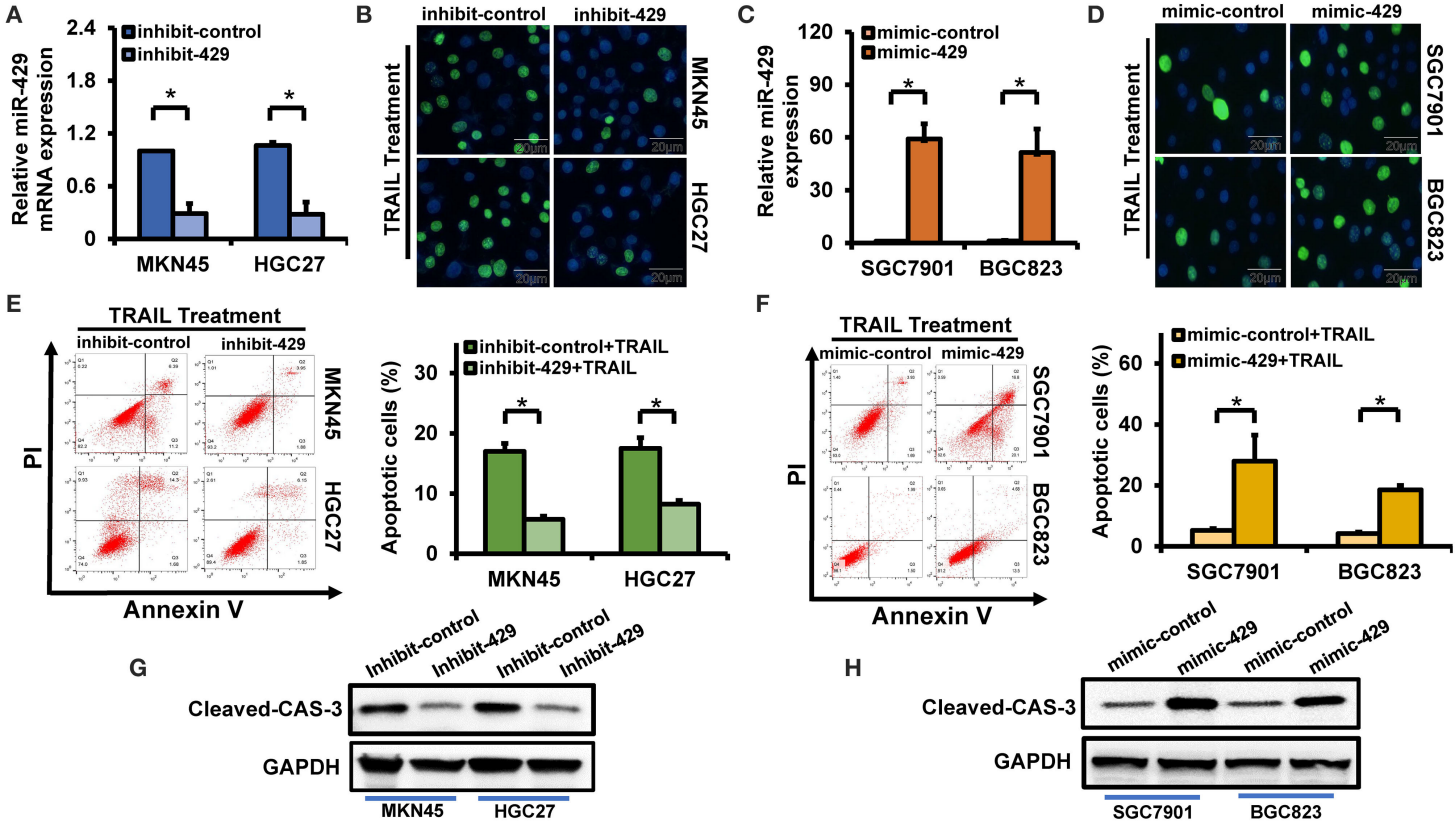
Relationship between miR-429 and gastric cancer (GCa) tumor necrosis factor-related apoptosis-inducing ligand (TRAIL) sensitivity. **(A)** Expression of miR-429 in cells transfected with inhibitors. **(B)** Apoptotic cells assessed by terminal deoxynucleotidyl transferase UTP nick-end labeling (TUNEL) staining following TRAIL treatment (100 ng/ml) in inhibitor-transfected cells. *Scale bar*, 25 μm. **(C)** Expression of miR-429 in cells transfected with mimics. **(D)** Mimic-429 was transfected into SGC7901 and BGC823 cells and apoptosis was assessed *via* TUNEL staining after TRAIL treatment (100 ng/ml). *Scale bar*, 25 μm. **(E)** Inhibitor-429 was transfected into the indicated cells and apoptosis was assessed with annexin V/propidium iodide (PI) staining after TRAIL treatment (100 ng/ml). **(F)** Mimic-429 was transfected into the indicated cells and apoptosis was assessed by flow cytometry after TRAIL treatment (100 ng/ml). **(G)** Inhibitor-429 was transfected into the indicated cells and cleaved caspase-3 expression was assessed by Western blot analysis after TRAIL treatment (100 ng/ml). **(H)** Mimic-429 was transfected into the indicated cells and cleaved caspase-3 expression was assessed by Western blot after TRAIL treatment (100 ng/ml). The results are presented as the mean ± SEM of triplicate samples from three independent experiments. *P < 0.05.

miR-429 is highly expressed in TRAIL-sensitive HGC27 and MKN45 cells. miR-429 inhibitors were next transfected to reduce miR-429 expression by ~70% (Figure 2A). Forty-eight hours post-transfection, the cells were TRAIL treated and the percentage of apoptotic cells was assessed *via* TUNEL assays. We found that HGC27 and MKN45 had increased sensitivity to TRAIL after transfection of the miR-429 inhibitor (Figure 2B). The apoptotic rates were then assessed by flow cytometry, in which significantly lower levels of apoptosis were observed following inhibitor treatment (Figure 2E). These findings were confirmed through the loss of cleaved caspase-3 expression following TRAIL treatment in cells inhibited by miR-429 (Figure 2G). The silencing of miR-429 therefore decreased TRAIL sensitivity in the GCa cell lines.

### miR-429 Mimics Sensitize GCa Cells to TRAIL

miR-429 is poorly expressed in BGC823 and SGC7901 cells, both of which are resistant to TRAIL. Following transfection with miR-429 mimics, its expression increased ~50-fold or more (Figure 2C). Following transfection, cells were TRAIL treated and the number of apoptotic cells were assessed *via* TUNEL assays and flow cytometry. Transfection of mimic-429 enhanced the TRAIL sensitivity of BGC823 and SGC7901 (Figures 2D,F), as evidenced by the enhanced expression of cleaved caspase-3 following TRAIL treatment (Figure 2H).

### Identifying the Cellular Targets of miR-429

**Figure 3:**
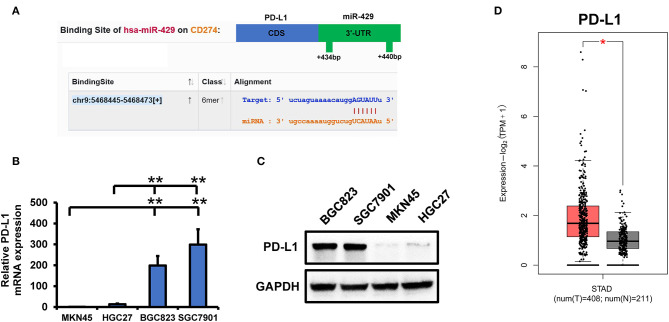
PD-L1 is targeted by miR-429 to mediate tumor necrosis factor-related apoptosis-inducing ligand (TRAIL) sensitivity. **(A)** Binding of miR-429 and PD-L1 predicted from starBase. The position of the 3′-UTR binding targets of miR-429 and PD-L1 were predicted on TargetScan. **(B)** mRNA expressions of PD-L1 in SGC7901, BGC823, MKN45, and HGC27 cells *via* quantitative PCR (qPCR). **(C)** Western blot analysis of PD-L1 expressions in SGC7901, BGC823, MKN45, and HGC27 cells. **(D)** Expressions of PD-L1 in tumor and normal tissues analyzed using the TCGA database on the GEPIA2 website. Results are the mean ± SEM of triplicate samples from three independent experiments. *P < 0.05, **P < 0.01.

To identify the miR-429 targets that mediate TRAIL sensitivity, we screened for conserved miR-429 targets on Targetscan. We previously identified changes in PD-L1 expressions in sensitive vs. resistant cells (Figures 3B,C). PD-L1 was also identified as a conserved target of miR-429 (Figure 3A). The expressions of PD-L1 in tumor and normal tissues in the GEPIA identified PD-L1 as highly expressed in tumor tissues (Figure 3D). PD-L1 was previously identified to play a role in tumor immunity and chemoresistance. PD-L1 is also associated with EMT and cancer stem cell processes, and EMT and cancer stem cells are closely related to chemoresistance (24). PD-L1 expression was consistently higher in resistant cells following TRAIL treatment, while no changes in the sensitive cells were observed (Supplementary Figure 1D). We reasoned that miR-429 regulates GCa TRAIL sensitivity by downregulating PD-L1 expression.

### miR-429 Binds to the 3′-UTR of PD-L1 and Downregulates PD-L1 Expression

**Figure 4:**
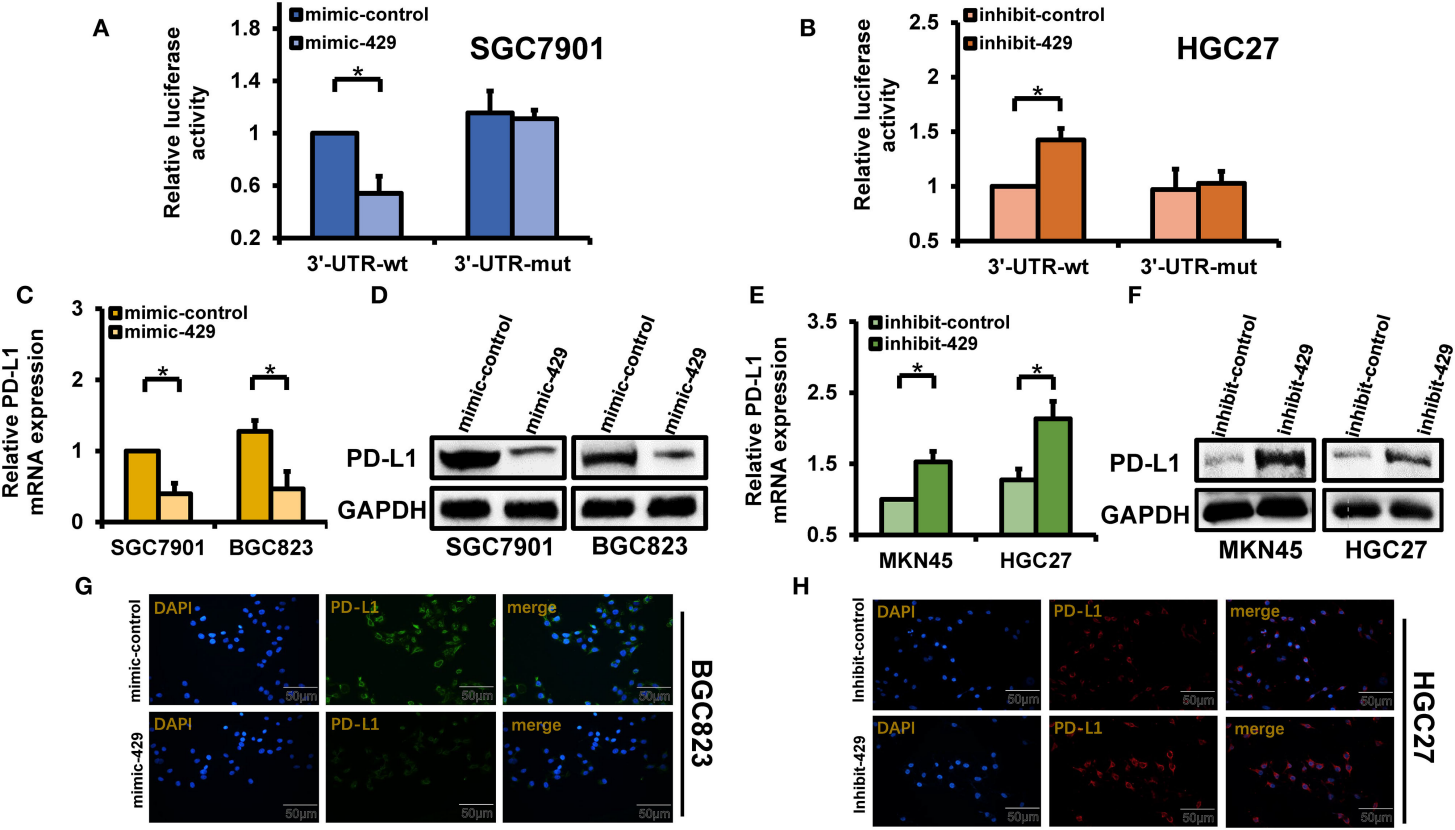
Regulatory relationship of miR-429 to PD-L1. **(A)** Cells were transfected with mimic-429 and PD-L1 luciferase reporter plasmids [wild type (WT) and mutant (MUT)] and dual-luciferase reporter assays were performed. **(B)** Luciferase reporter plasmids (WT and MUT) of inhibitor-429 and PD-L1 were transfected into HGC27 cells. Luciferase activity was assessed *via* dual-luciferase reporter assays. **(C)** Mimic-429 was transfected into SGC7901 and BGC823 cells and the expression of PD-L1 was detected by quantitative PCR (qPCR). **(D)** Mimic-429 was transfected into SGC7901 and BGC823 cells and the expression of PD-L1 was assessed *via* Western blot. **(E)** Inhibitor-429 was transfected into MKN45 and HGC27 cells and the mRNA expression of PD-L1 was assessed by qPCR. **(F)** Cells were transfected with inhibitor-429 and the expression of PD-L1 was assessed by Western blot. **(G)** Mimic-429-expressing BGC823 cells were assessed for PD-L1 localization and expression by immunofluorescence analysis. *Scale bar*, 50 μm. **(H)** Inhibitor-429 was transfected into HGC27 cells and the intracellular localization and expression of PD-L1 were assessed by immunofluorescence analysis. *Scale bar*, 50 μm. Results are the mean ± SEM of triplicate samples from three independent experiments. *P < 0.05.

The 3′-UTR of PD-L1 [wild type (WT) and mimics] and mimic-429 were transfected into SGC7901 cells that possess low endogenous levels of miR-429. Dual-luciferase reporter assays showed that the overexpression of miR-429 reduced the activity of WT 3′-UTR luciferase, while no changes occurred for the mutants (Figure 4A). Transfection of the 3′-UTR luciferase plasmids of the PD-L1 and miR-429 inhibitors into HGC27 cells increased the activity of the WT 3′-UTR luciferase, while no changes in the expression of the mutant were observed (Figure 4B). These data confirmed that the 3′-UTR of PD-L1 is a cellular target of miR-429. The transfection of mimic-429 into SGC7901 and BGC823 cells was used to detect changes in PD-L1 expression, which significantly decreased (Figures 4C,D). Inhibitor-429, when transfected into HGC27 and MKN45 cells, also increased PD-L1 expression (Figures 4E,F). These data confirm that miR-429 regulates the expression of PD-L1 at the mRNA and protein levels. Immunofluorescence assays were also used to confirm the localization of PD-L1 in cells transfected with mimics and inhibitors of miR-429. The results showed that the mimics downregulated the expression of PD-L1 (Figure 4G) while the inhibitors produced the opposing phenotype (Figure 4H). In summary, the expressions of miR-429 and PD-L1 led to a significant negative regulatory relationship.

### PD-L1 Is Targeted by miR-429

**Figure 5:**
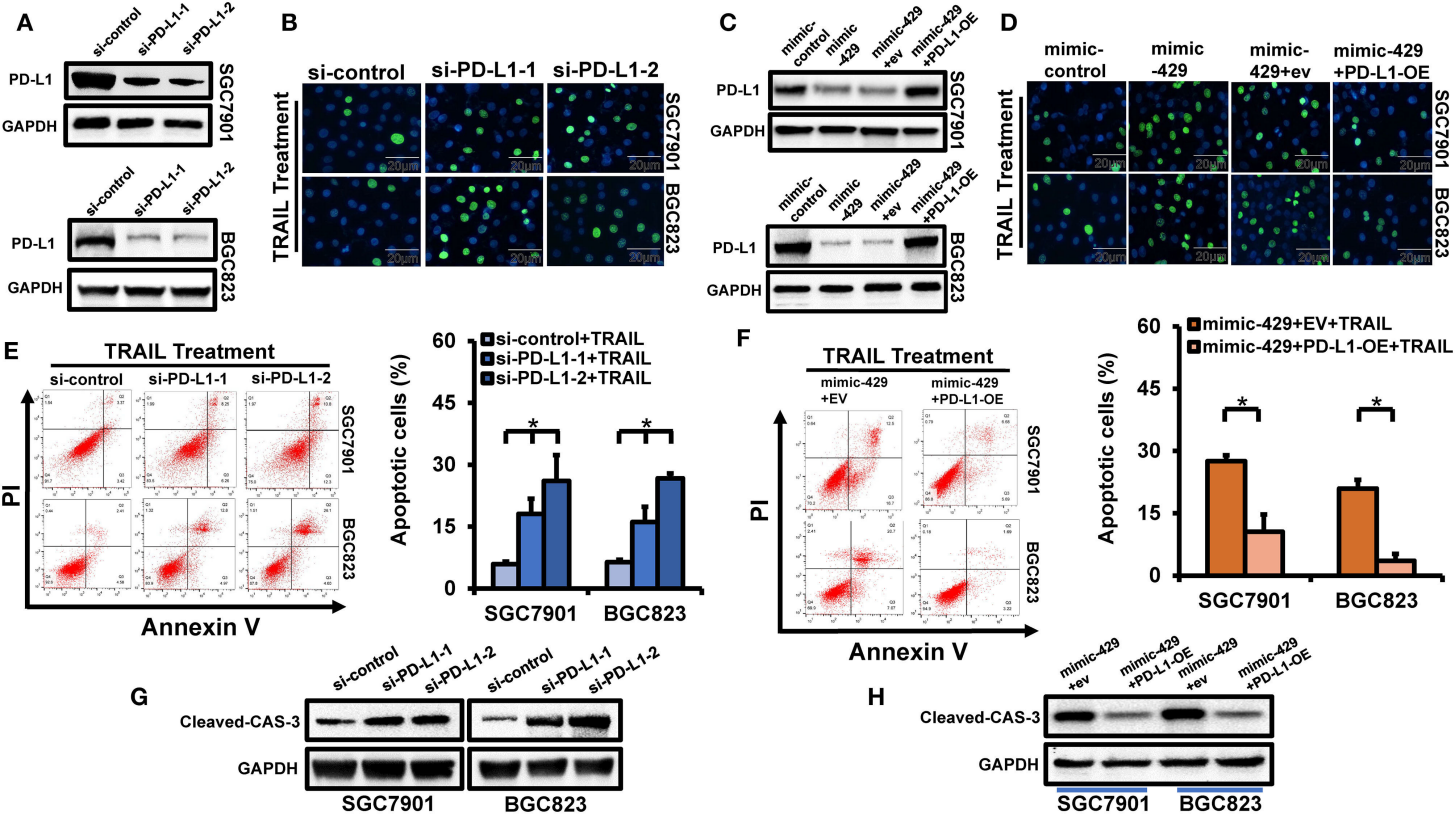
Effects of PD-L1 on tumor necrosis factor-related apoptosis-inducing ligand (TRAIL) sensitivity in gastric cancer (GCa) cells. **(A)** Cells were transfected with si-PD-L1 and PD-L1 expression was assessed by Western blot analysis. **(B)** Cells were transfected with si-PD-L1 and the number of apoptotic cells were assessed by terminal deoxynucleotidyl transferase UTP nick-end labeling (TUNEL) staining after TRAIL treatment (100 ng/ml). *Scale bar*, 25 μm. **(C)** Cells were transfected with mimic-429, mimic-control, empty vector (EV), and PD-L1-OE (PD-L1 cDNA) lacking the 3′-UTR to rescue PD-L1 expression. The PD-L1 levels were assessed by Western blot. **(D)** SGC7901 and BGC823 cells were co-transfected with mimic-429, mimic-control, EV, and PD-L1-OE (PD-L1 cDNA) without the 3′-UTR and the proportion of apoptotic cells were assessed by TUNEL staining after TRAIL treatment (100 ng/ml). *Scale bar*, 25 μm. **(E)** SGC7901 and BGC823 cells were transfected with si-PD-L1 and annexin V/propidium iodide (PI) stained to detect apoptotic cells by flow cytometry after TRAIL treatment (100 ng/ml). **(F)** SGC7901 and BGC823 cells were co-transfected with mimic-429, EV, and PD-L1-OE (PD-L1 cDNA) without the 3′-UTR and annexin V/PI stained to detect apoptosis by flow cytometry after TRAIL treatment (100 ng/ml). **(G)** SGC7901 and BGC823 cells were transfected with si-PD-L1 and the expression of cleaved caspase-3 was assessed *via* Western blot analysis after TRAIL treatment (100 ng/ml). **(H)** SGC7901 and BGC823 cells were co-transfected with mimic-429, EV, and PD-L1-OE (PD-L1 cDNA) lacking the 3'-UTR and the expression of cleaved caspase-3 was detected by Western blot after TRAIL (100 ng/ml) treatment. Results are the mean ± SEM of triplicate samples from three independent experiments. *P < 0.05.

miR-429 influences the sensitivity of GCa cells to TRAIL through PD-L1. Whether PD-L1 expression directly regulates TRAIL sensitivity has not been verified in detail. We screened two siRNAs for PD-L1 expression, which significantly declined in BGC823 and SGC7901 cells that overexpress PD-L1 (Figure 5A). After silencing PD-L1, the GCa cells treated with TRAIL showed significantly increased rates of apoptosis (Figures 5B,E), confirmed by the increased levels of cleaved caspase-3 (Figure 5G). To further confirm whether miR-429 regulates the sensitivity of GCa cells to TRAIL, the expression of PD-L1 was assessed through rescue experiments. The PD-L1 coding region (CDS) lacking a 3′-UTR was transfected into BGC823 and SGC7901 cells with mimic-429; loss of PD-L1 expression was observed (Figure 5C). TUNEL assays showed that the restoration of PD-L1 expression after transfection of mimic-429 in TRAIL-resistant cells restored their resistance (Figures 5D,F). Western blot analysis for cleaved caspase-3 also showed that the overexpression of PD-L1 restored TRAIL resistance (Figure 5H). Taken together, these data demonstrate that miR-429 regulates the sensitivity of GCa cells to TRAIL through its regulation of PD-L1, consistent with the regulation of PD-L1 expression by miR-429.

### PD-L1 Maintains Cell Viability and Survival Through EGFR

**Figure 6:**
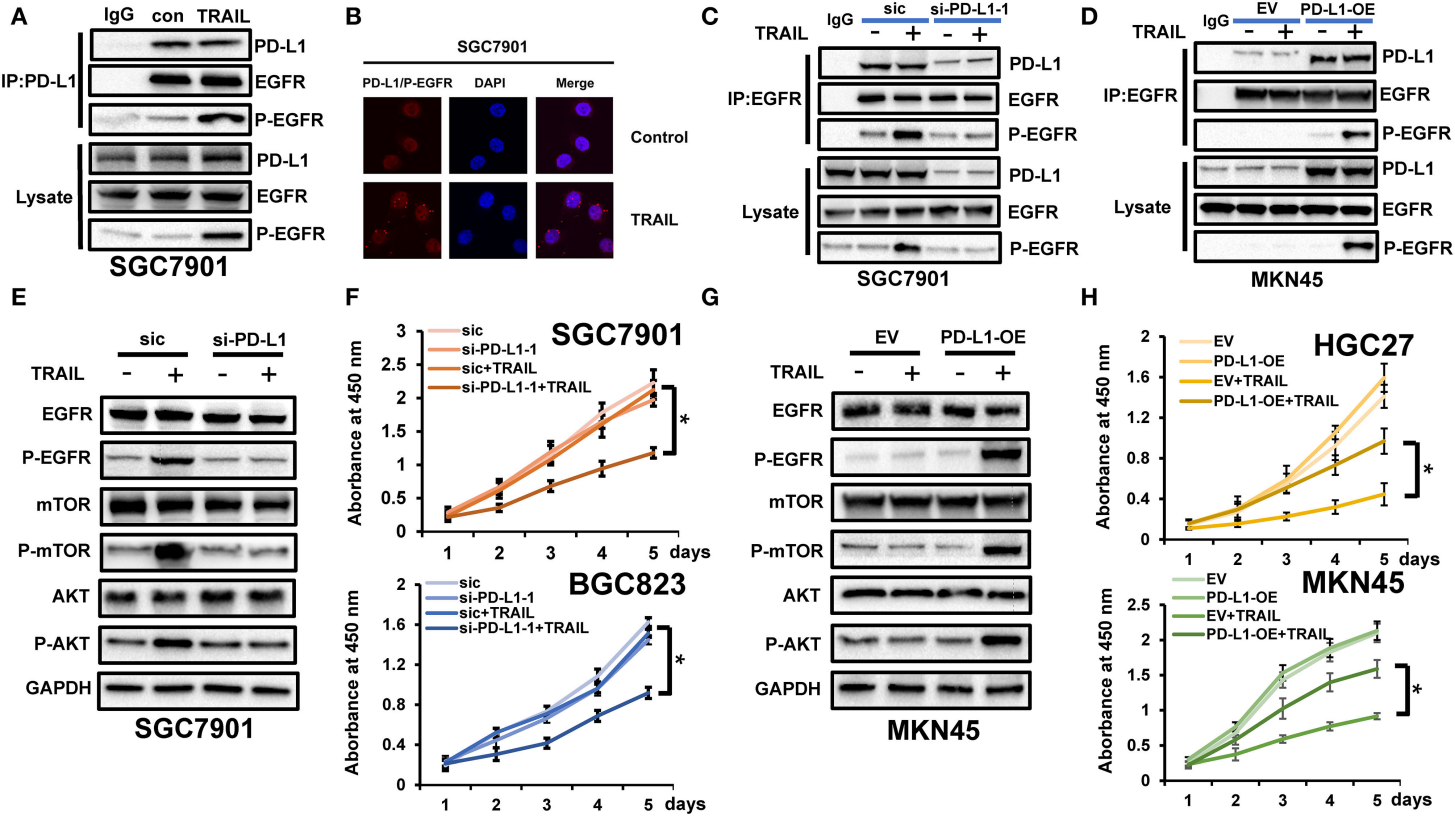
PD-L1 maintained cell viability and survival through epidermal growth factor receptor (EGFR) binding. **(A)** Immunoprecipitation (IP) using anti-PD-L1 antibodies to detect PD-L1 binding to EGFR and phosphorylated EGFR (p-EGFR) in SGC7901 cells after tumor necrosis factor-related apoptosis-inducing ligand (TRAIL) treatment (100 ng/ml). **(B)** Interactions of PD-L1 and p-EGFR in SGC7901 cells analyzed by Duolink *in situ* proximity ligation assay (PLA) after TRAIL treatment (100 ng/ml). *Red*, interaction of PD-L1 and p-EGFR; *blue*, cell nuclei stained with DAPI (magnification, ×60). **(C)** PD-L1 was silenced in SGC7901 cells and the expressions of PD-L1 and p-EGFR were assessed by immunoprecipitation assays using anti-EGFR antibodies following TRAIL treatment (100 ng/ml). **(D)** PD-L1 is overexpressed in MKN45 cells. Expressions of PD-L1 and p-EGFR were assessed using anti-EGFR IP following TRAIL treatment (100 ng/ml). **(E)** PD-L1 silencing in SGC7901 cells and AKT/mTOR assessments in cells treated with TRAIL (100 ng/ml). **(F)** Si-PD-L1 was transfected into SGC7901 and BGC823, cells were treated with TRAIL (100 ng/ml), and cell viability was assessed. **(G)** PD-L1-OE was transfected into MKN45 cells and AKT/mTOR activation was assessed following Western blot analysis after TRAIL treatment (100 ng/ml). **(H)** Cells were transfected with PD-L1-OE and cell viability was assessed following TRAIL treatment (100 ng/ml). Results are presented as the mean ± SEM of triplicate samples from three independent experiments. *P < 0.05.

PD-L1 regulates the malignant phenotypes of tumor cells. In our previous studies, TRAIL treatment in TRAIL-resistant cells promoted the phosphorylation of EGFR and maintained cell viability to counteract TRAIL-induced apoptosis (Figure 6A) (15). To verify whether PD-L1 interacts with EGFR, we used SGC7901 to carry out co-IP experiments. The results showed that PD-L1 can combine with EGFR, and EGFR activation to p-EGFR increased significantly under TRAIL treatment (Figure 6A). At the same time, we used SGC7901 to carry out *in situ* proximity restriction assays. The results showed that PD-L1 and p-EGFR had protein interactions under TRAIL treatment (Figure 6B). We speculated that PD-L1 mediates TRAIL resistance through its effects on EGFR. Following PD-L1 silencing, SGC7901 cells were TRAIL treated and the levels of EGFR and p-EGFR were assessed. While no changes in the total EGFR expression were observed, the levels of p-EGFR declined (Figure 6C). Similarly, the overexpression of PD-L1 in TRAIL-sensitive MKN45 cells did not influence the EGFR expression, but enhanced the levels of p-EGFR (Figure 6D). We therefore conclude that PD-L1 binding to EGFR mediates TRAIL sensitivity in GCa cells. We further examined the effects of PD-L1 on mTOR and AKT downstream of p-EGFR. After silencing PD-L1 in SGC7901 cells treated with TRAIL, the activation of mTOR and AKT decreased (Figure 6E). In contrast, PD-L1 overexpression in MKN45 cells following TRAIL treatment increased the levels of mTOR and AKT compared to the control cells (Figure 6G). These data highlighted the role of PD-L1 in EGFR activation and TRAIL resistance. We further verified that PD-L1 regulates the viability of GCa cells following TRAIL activation in the cell viability assays. As PD-L1 expressions in TRAIL-resistant cells BGC823 and SGC7901 decreased, loss of cell viability was observed following TRAIL treatment (Figure 6F). Upon the overexpression of PD-L1 in TRAIL-sensitive HGC27 and MKN45 cells, the cell viability decreased following TRAIL treatment (Figure 6H). PD-L1 therefore contributes to GCa resistance to TRAIL.

## Discussion

Although GCa cells are resistant to TRAIL, it represents a promising tumor treatment due to its tumor killing selectivity and low levels of toxicity to healthy tissues. Revealing the mechanism of GCa resistance to human recombinant TRAIL therefore provides a theoretical basis for its clinical application (7).

Lipid rafts provide a platform for DR4 and DR5 to facilitate the assembly of death-inducing signaling complex (DISC) leading to apoptosis (15, 22, 25, 26). However, we found that TRAIL treatment led to the relocalization of EGFR to lipid rafts, leading to its activation and TRAIL resistance. Inhibiting EGFR further enhanced the effects of TRAIL on GCa cells (15). In addition, we found that the downregulation of the cbl family upregulated the sensitivity of gastric cancer cells to TRAIL. We screened the miRNA expressions in TRAIL-resistant and TRAIL-sensitive cells using Affymetrix miRNA microarrays. A high differential expression was observed in the miR-429/miR-200 family, which we verified with the dual-luciferase reporter assays. miR-429/miR-200 do not regulate cbl, and we hypothesize that miR-429/miR-200 may affect sensitivity to TRAIL through the modulation of other molecules (22).

We found that DR4, DR5, and PD-L1 are targets of miR-429/miR-200 on Targetscan. We further verified the expressions of these genes in GCa cells. No significant difference in the expressions of DR4 and DR5 in sensitive and resistant cells were observed, while PD-L1 was highly expressed in resistant cells. Meanwhile, we determined that miR-429 had no regulatory effect on DR4 and DR5 (Supplementary Figure 1B). Most PD-L1 studies have focused on its role in immunosuppression and immunotherapy. PD-L1 exerts immunosuppressive effects by binding to PD-1 ligands on immune cells *in vivo*, promoting immune escape (27). Recent studies indicate an intrinsic role for PD-L1 in modulating EMT (28, 29), cancer stem cell (CSC)-like phenotypes (30, 31), and metastasis and resistance to therapy (24). In addition, the miR-429/miR-200 expressions strongly correlated with the EMT processes (32). These malignant phenotypes led to the association of PD-L1 with tumor cell resistance (33), including bladder carcinoma (34), non-small-cell lung cancer (35), breast cancer (17, 36), and head and neck squamous cell carcinoma (37). This study revealed the role ofPD-L1 in tumorigenesis during TRAIL resistance.

One of the most important cell survival receptors is the EGFR. TRAIL receptors (TRAIL-Rs) are key to the regulation of tumor apoptosis. EGFRs activate the survival pathways, including PI3K/AKT, Ras/MAPK, and JAK/STAT signaling. TRAIL activates the apoptotic signaling pathways, leading to caspase, and mitochondrial activation. The balance between these signaling pathways determines the cell viability and apoptotic status (38). We simultaneously found that TRAIL could induce EGFR activation, leading to TRAIL resistance. PD-L1 and EGFR interact in colon cancer cells, and their combination in GCa may influence their sensitivity to TRAIL. Since the miR-200 target sequences are identical, we selected miR-429, which had the highest differential expression. The results showed that miR-429 could affect the sensitivity of GCa cells to TRAIL through targeting PD-L1. We explored the mechanism of PD-L1 binding to EGFR, which promotes GCa resistance by regulating the phosphorylation of EGFR, leading to maintaining the cell viability. In addition, the miR-429/miR-200 family regulates EGFR expression (39), which may further enhance the anti-TRAIL effects of PD-L1 in combination with EGFR. Lipid rafts regulate the sensitivity of GCa cells to TRAIL (22). However, whether PD-L1 and miR-429 regulate lipid rafts has not been verified. PD-L1 may bind to EGFR and promote EGFR activation in lipid rafts. At the same time, PD-L1 expression influences the mutational status of EGFR (40). Thus, other effects on the sensitivity of GCa to TRAIL cannot be dismissed.

The effects of TRAIL/TRAIL-R activation on PD-L1 expression were further verified. Interestingly, miR-429 expressions in TRAIL-resistant cells decreased following TRAIL treatment, while PD-L1 increased TRAIL resistance. However, this phenomenon did not occur in TRAIL-sensitive cells. We hypothesized that TRAIL regulates the expression of miR-429 to further regulate PD-L1 expression. This may be related to TRAIL/TRAIL-R-mediated non-apoptotic signals (41). TRAIL/TRAIL-R signaling did not influence the expression of PD-L1. TRAIL-R is not only expressed on the surface of tumor cells but is also widely distributed on the surface of various immune cells. Whether the effects of TRAIL/TRAIL-R activation on the tumor immune microenvironment were positive or negative remains unclear (42). In addition, the effects of PD-1/PD-L1 activation in tumor cells on TRAIL sensitivity have not been investigated. Whether PD-1/PD-L1 and TRAIL/TRAIL-R interact in the tumor microenvironment requires further analysis. *In vivo* experiments assessing PD-1 were not performed, and we will continue to explore these in future studies.

**Figure 7:**
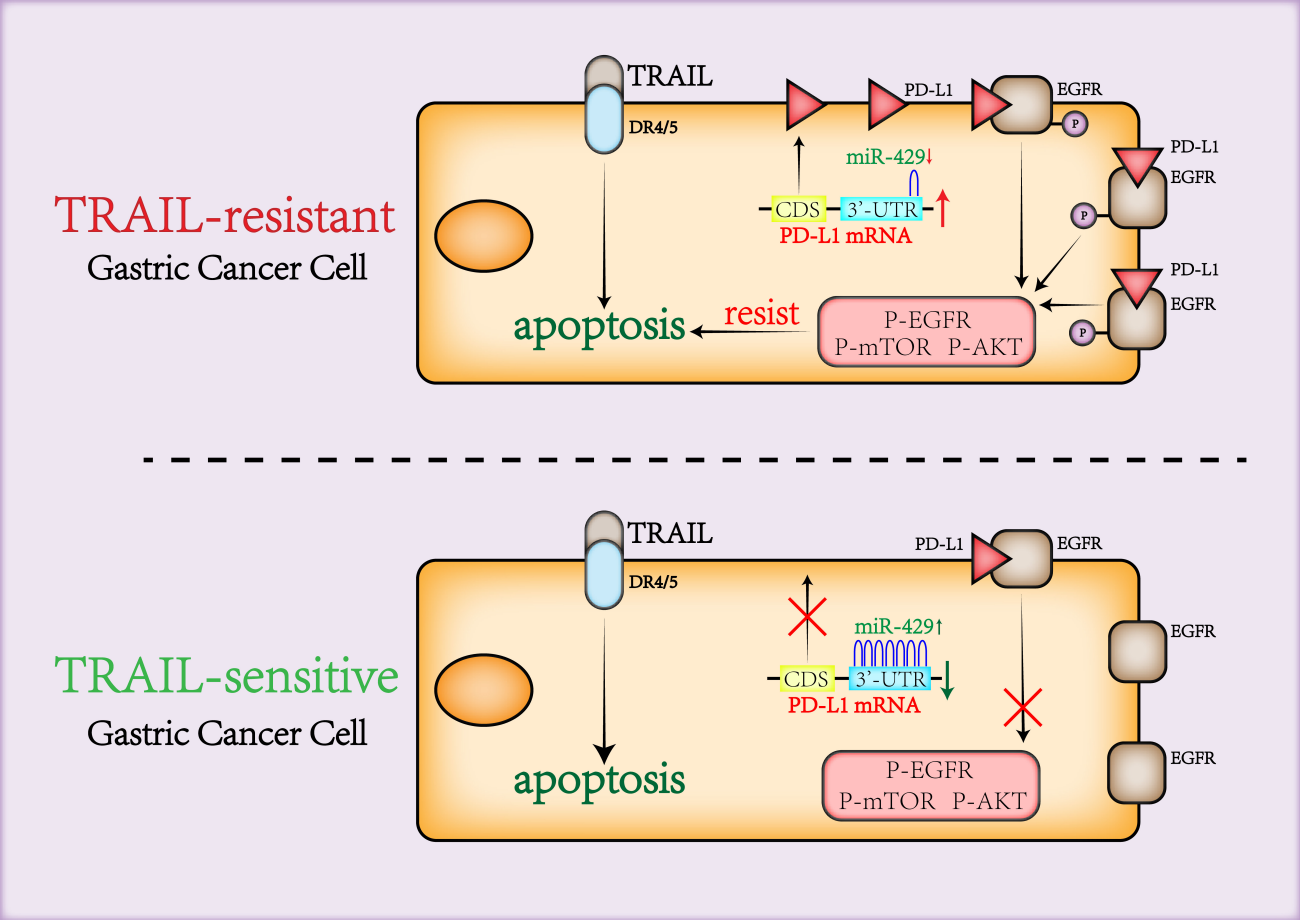
A working model of miR-429/PD-L1/EGFR axis in gastric cancer sensitivity to TRAIL.

In summary, we report that PD-L1 maintains cell viability through binding to and activating EGFR under the regulation of miR-429 to mediate TRAIL sensitivity in GCa cells (Figure 7). The combination of TRAIL and trastuzumab significantly enhanced its anti-tumor effects (43). Although further validation in *in vivo* experiments is required, this study provides a new theoretical basis for the combination of PD-L1 inhibitors and recombinant TRAIL in GCa and increases the screening of patients who are sensitive to TRAIL treatment.

## Data Availability Statement

The datasets presented in this study can be found in online repositories. The names of the repository/repositories and accession number(s) can be found below: Array express database (https://www.ebi.ac.uk/arrayexpress/; Accession: E-MTAB-9151).

## Author Contributions

LX and XQ designed the research. JL and TG performed the data acquisition. XC and CL supervised the data and algorithms. SW and JG performed data analysis and interpretation. PW and YL carried out the statistical analysis. JL prepared the manuscript. LX and XQ participated in manuscript editing and review. All authors read and approved the final manuscript.

## Conflict of Interest

The authors declare that the research was conducted in the absence of any commercial or financial relationships that could be construed as a potential conflict of interest.
