# Supplementary figures and images for: PD-L1 Under Regulation of miR-429 Influences the Sensitivity of Gastric Cancer Cells to TRAIL by Binding of EGFR
Source: Front Oncol. 2020 Jul 22;10:1067. doi: 10.3389/fonc.2020.01067 (PMC7387728; doi:10.3389/fonc.2020.01067)

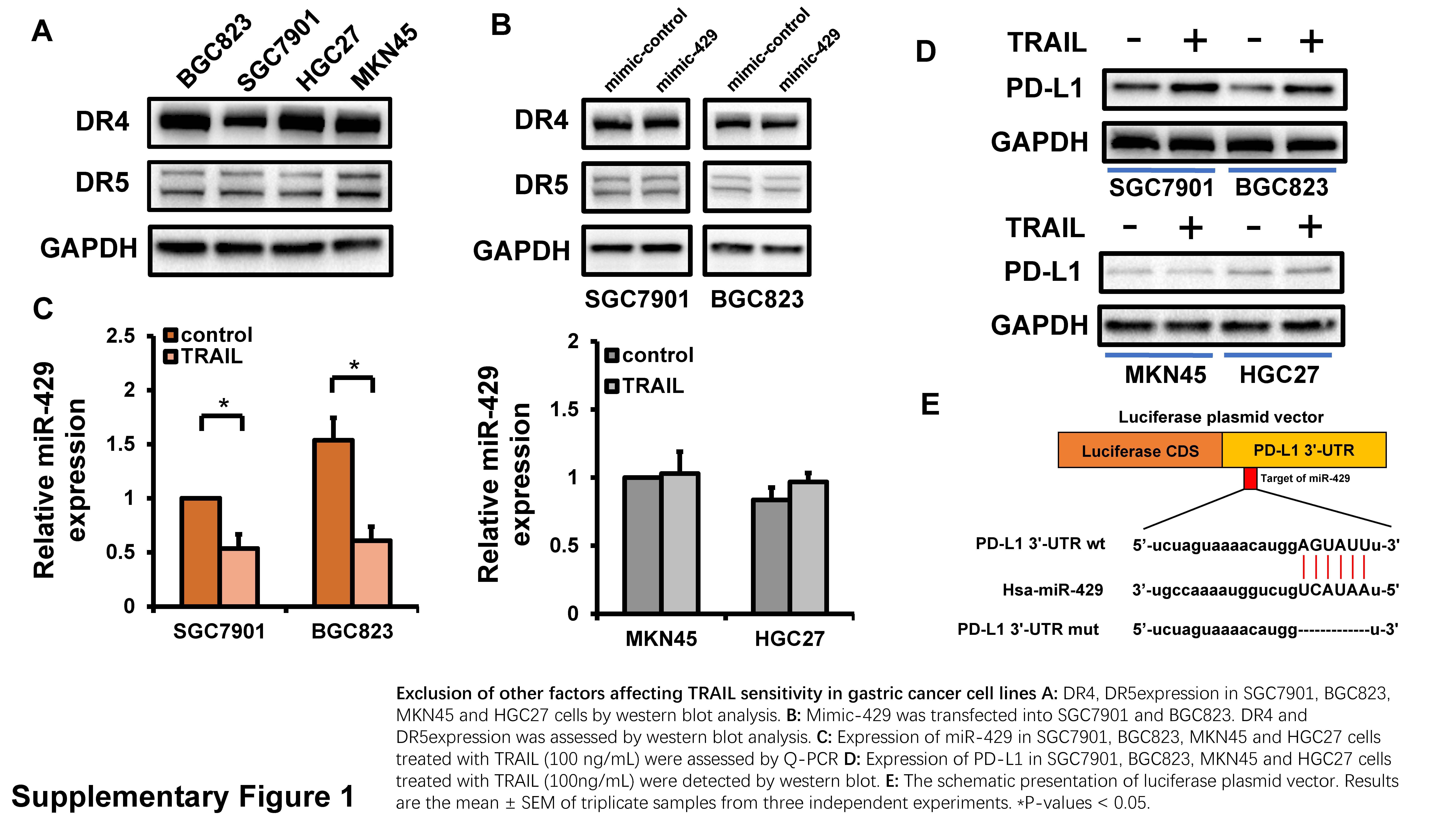

Supplement: Supplementary file 3 [file Image_1.tif]
